# Supplementary material for: KLF5 enables dichotomous lineage programs in pancreatic cancer via the AAA+ ATPase coactivators RUVBL1 and RUVBL2
Source: Nat Commun. 2025 Nov 15;16:9996. doi: 10.1038/s41467-025-66007-0 (PMC12619835; doi:10.1038/s41467-025-66007-0)
Supplement: Supplementary file 1 — Supplementary Information [file 41467_2025_66007_MOESM1_ESM.pdf]

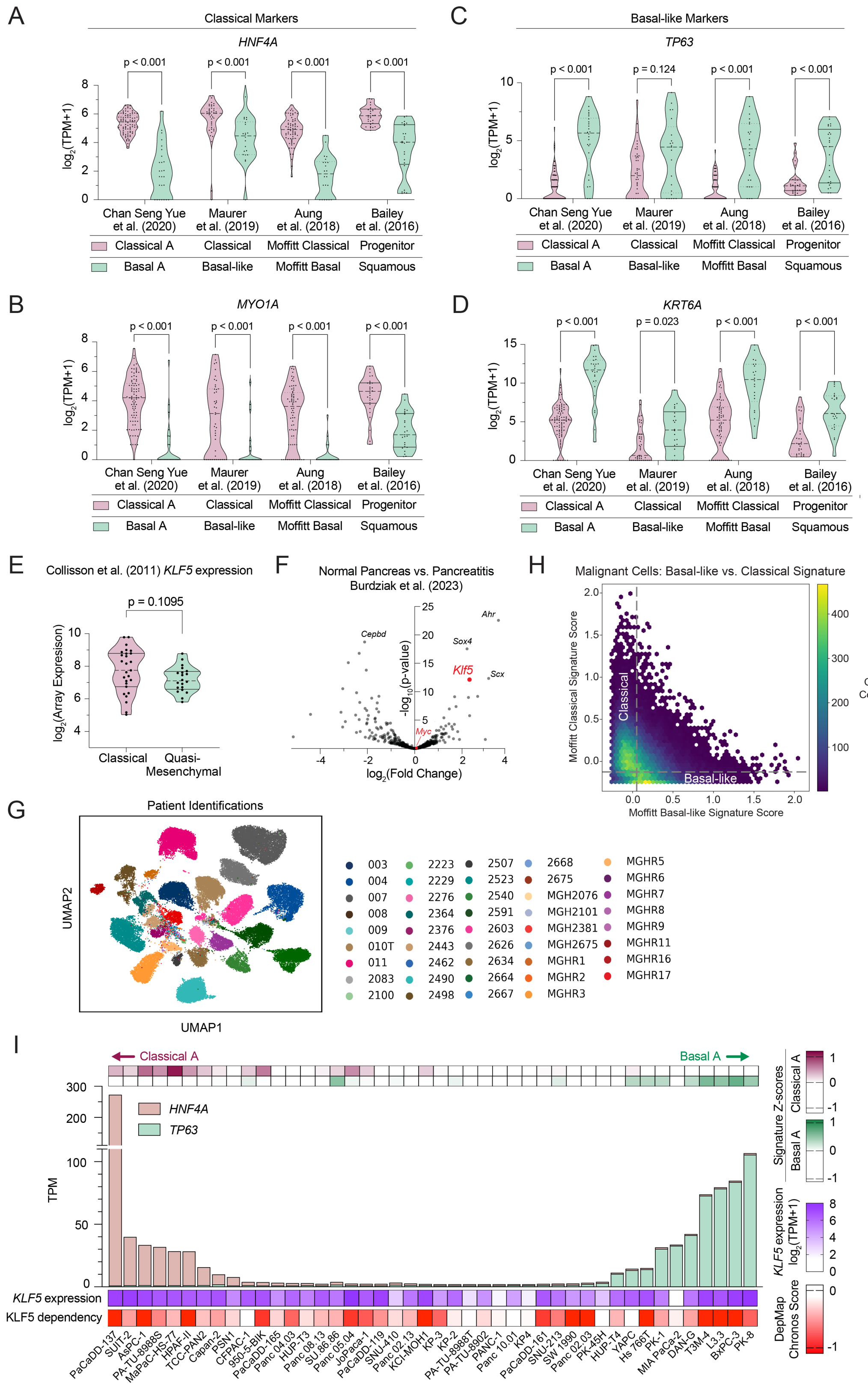

Supplementary Figure 1. Characterization of the classical and basal-like subtypes of human PDAC. (A-E) Gene expression in resected human PDAC tissue. Significance evaluated using a two-

tailed Mann–Whitney test. (A-D) RNA-seq data reanalyzed from Chan Seng Yue et al. 2020, Maurer et al. 2019, Aung et al. 2018, or Bailey et al. 2016. Individual samples were assigned to Classical/Progenitor or Basal/Squamous according to the classifications in each respective study. Gene expression (log_2(TPM+1)) is plotted. Multiple comparison correction for 4 comparisons. See also Supplementary Data 2. (E) *KLF5* microarray expression was reanalyzed from Collisson et al. 2011. Samples assigned according to the published subtypes. (F) Single cell RNA-sequencing of mouse pancreatic epithelial cells reanalyzed in bulk from Burdziak et al. 2023. Volcano plots of differential expression of 642 transcription factors in normal pancreatic epithelial cells versus collected 48-hours post cerulein treatment (pancreatitis induction). n = 2 samples per condition. Fold change and significance calculated by DESeq2. (G-H) Single nucleus RNA-sequencing (snRNA-seq) from 43 resected human PDAC tumors reanalyzed from Hwang et al. 2022. (G) UMAP of malignant nuclei (dots), colored by Patient ID. (H) Designation of Classical and Basal classifications for malignant cells. Each cell was scored based on the mean normalized expression score across all genes in the Moffitt classical or basal-like gene set. Each dot represents a cell or group of cells, colored by cell density. Classical_score = -0.125 and basal_score = 0.05 were used as cutoffs to define Classical and Basal-like populations, labeled on the graph in the top left and lower right quadrants respectively. (I) Gene expression in human PDAC cell lines (CCLE). Bar chart shows *HNF4A* and *TP63* expression in each cell line, ordered by *HNF4A* expression – *TP63* expression. Heatmap (top) shows median expression of Basal-A or Classical-A identity signature genes (Chan Seng Yue et al. 2020; Z-score analysis of variance-stabilized transformed counts across cell lines). Scale bars indicate Z-scores. Heatmap (middle) shows *KLF5* expression (log_2(*KLF5* TPM+1)). Heatmap (bottom) shows KLF5 dependency (Chronos score, DepMap). See also Supplementary Data 3. Source data are provided as a Source Data file.

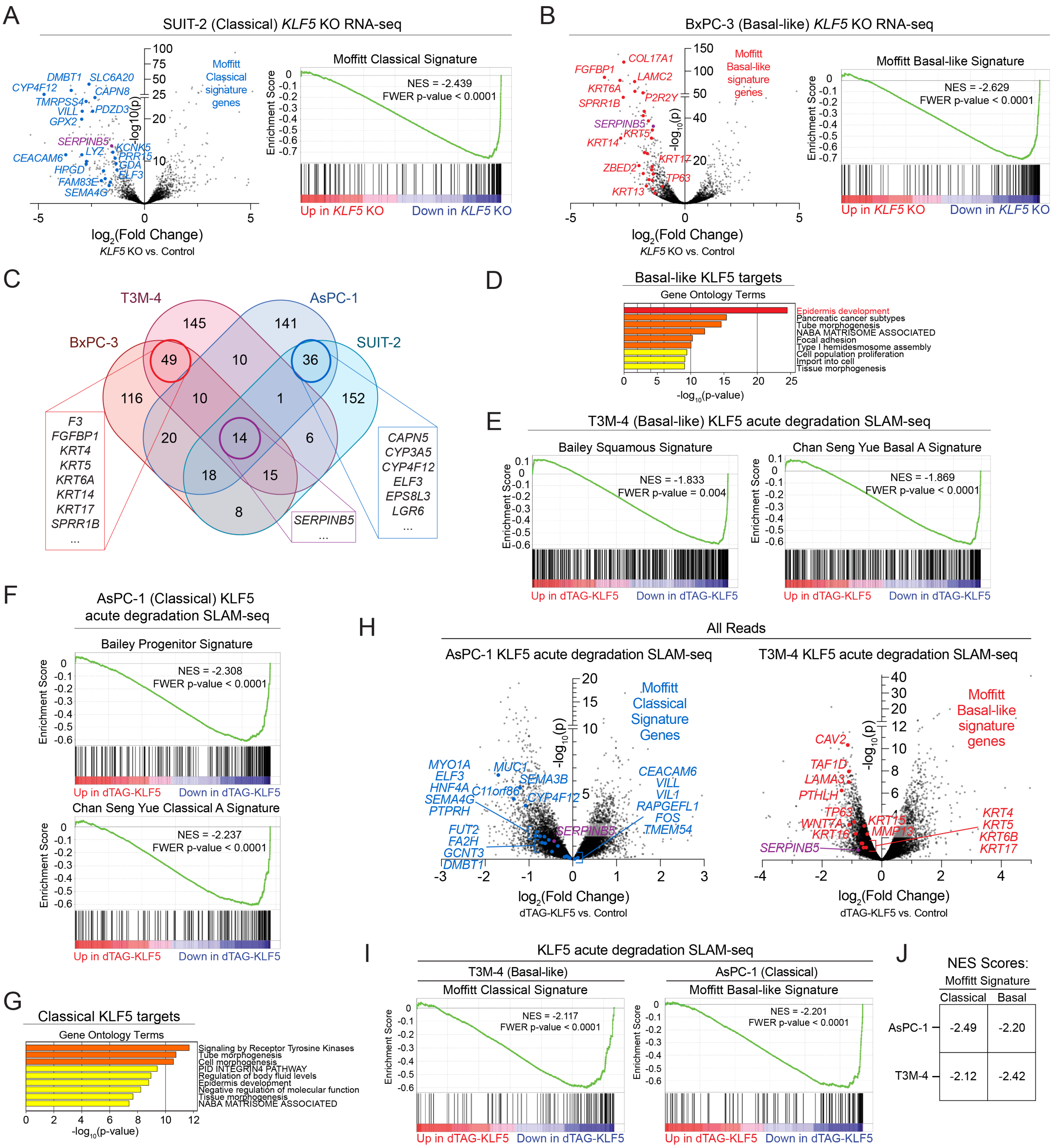

Supplementary Figure 2. KLF5 differentially regulates classical and basal identity genes in PDAC. (A-C) RNA sequencing in AsPC-1, SUIT-2, BxPC-3, and T3M-4 cells on day 5 following CRISPR-Cas9 knockout (KO) of *KLF5* or *ROSA26* (control). Fold change and significance calculated by DESeq2. Two independent sgRNAs were used for each KO. n = 4 biological replicates per cell line. See also Supplementary Data 4. (A-B) Volcano plots of differentially expressed genes following *KLF5* KO. Select genes within the Classical and Basal-like gene sets (Moffitt et al. 2015) and *SERPINB5* are labeled. Gene set enrichment analysis of differentially expressed genes following

KLF5 KO. Significance calculated by GSEA. NES = Normalized Enrichment score. FWER = Family-wise Error Rate. (C) 4-way Venn diagram of top 250 differentially downregulated genes following *KLF5* KO in each cell line. Selected genes are labeled according to their overlap. (D-J) SLAM-seq in (D-E,H-J) T3M-4 or (F-J) AsPC-1 following 4-hour total treatment with 300nM dTAG^{v}-1 or dTAG^{v}-1-NEG (control), including 2-hour 4sU labeling. Representative of 2 (T3M-4) or 3 (AsPC-1) independent experiments. Fold change and significance of T→C converted transcripts following KLF5 degradation calculated by DESeq2. See also Supplementary Data 5. (D,G) Metascape Gene Ontology analysis of significantly downregulated transcripts following KLF5 degradation in (D) T3M-4 or (G) AsPC-1. Terms are ranked by their significance (p-value) and the most significant terms ($-\log_{10}(p) > 10$) are highlighted. (E-F, I-J) Gene set enrichment analysis of differentially expressed transcripts following KLF5 degradation. Significance calculated by GSEA. NES = Normalized Enrichment score. FWER = Family-wise Error Rate. (H) Volcano plots of differentially expressed transcripts following *KLF5* degradation. Select genes within the Classical and Basal-like gene sets (Moffitt et al. 2015) and *SERPINB5* are labeled. All reads in the sequencing dataset are included in the analysis. Source data are provided as a Source Data file.

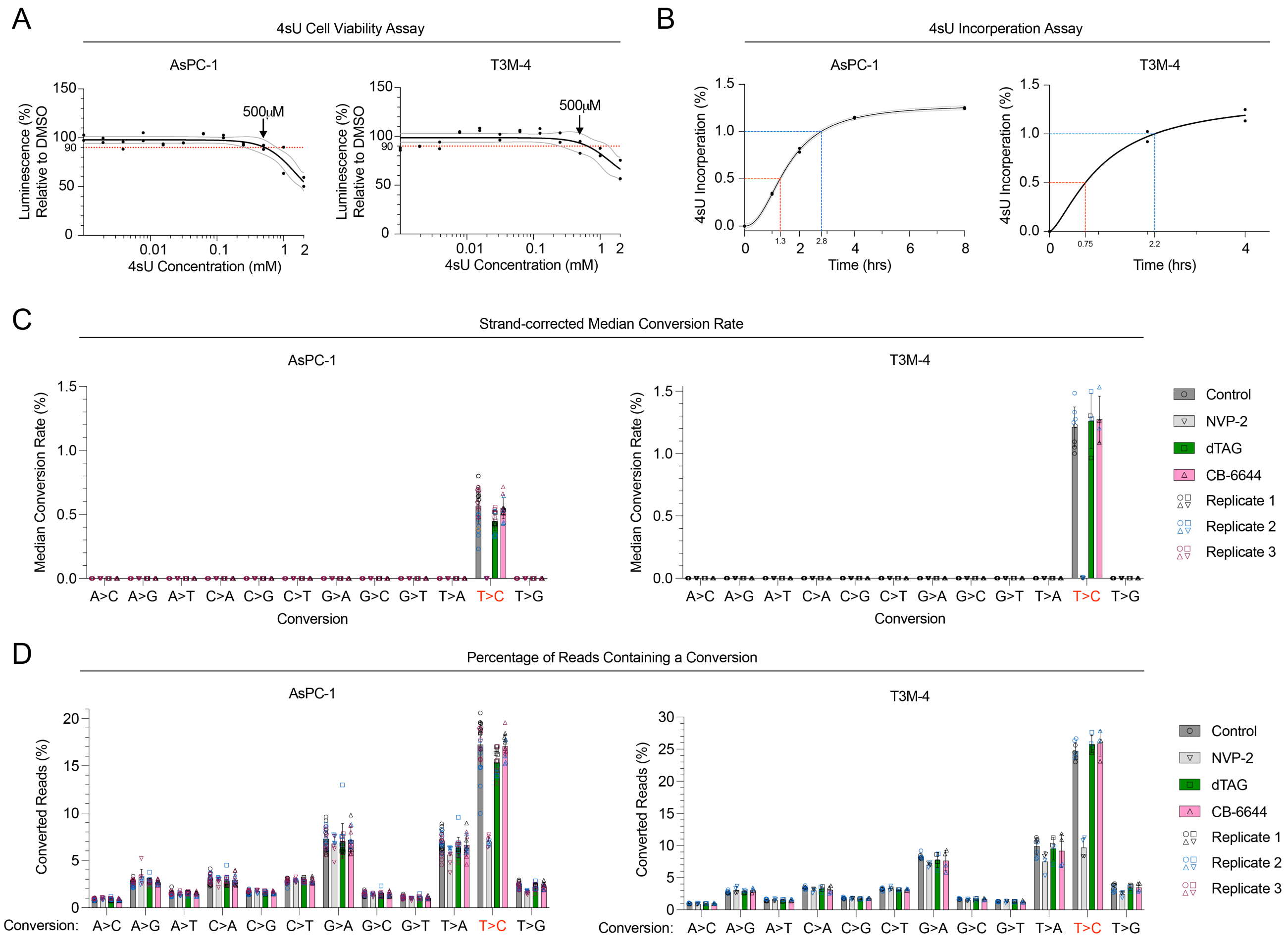

Supplementary Figure 3. Optimization and validation of the SLAM-seq assay. (A) CellTiter-Glo analysis in AsPC-1 and T3M-4 8 hours following treatment with 4sU. Luminescence of each 4sU treated sample was normalized to the luminescence of the DMSO treated sample. Two technical replicates are plotted for each cell line. Black line = sigmoidal interpolation ± 95% confidence interval. Red line = 90% of DMSO. (B) HPLC analysis of 4sU incorporation in AsPC-1 and T3M-4. Points indicate the abundance of identified 4sU nucleosides, relative to U nucleosides. Two technical replicates are plotted for each cell line. Black line = sigmoidal interpolation, Blue line = 1%, Red line = 0.5%. (C-D) (C) Median Conversion rates and (D) Percentage of called reads containing a conversion for each nucleotide combination in the AsPC-1 and T3M-4 SLAM-seq datasets. Each point is one sequencing sample. Median Conversion Rate is plotted as a percentage of all sequenced bases at each position for that sample, corrected for strand. Bars represent the mean ± SD. Source data are provided as a Source Data file.

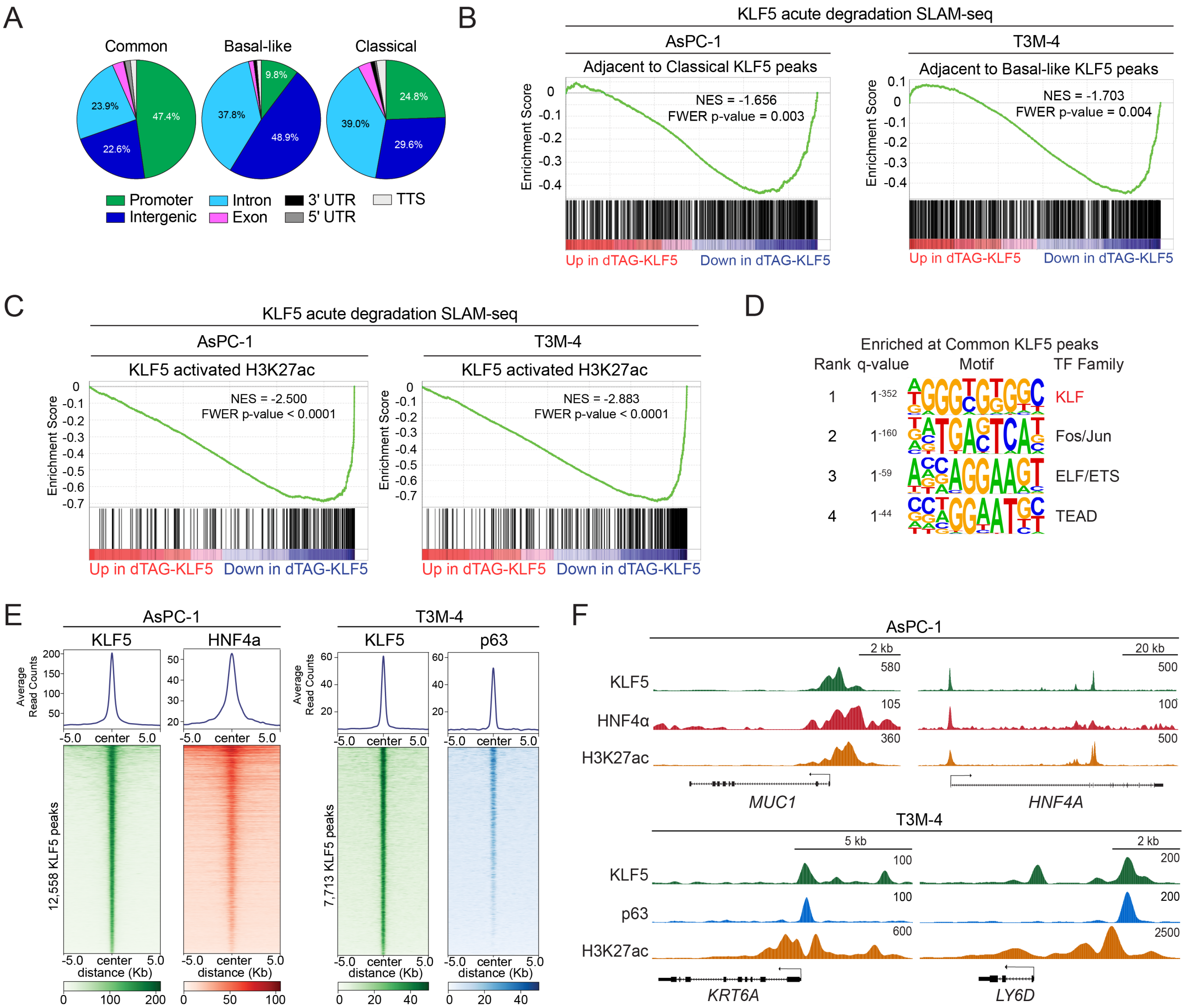

Supplementary Figure 4. KLF5 co-occupies chromatin with lineage specific master regulators in classical and basal-like PDAC. (A) HOMER annotations for common-, basal-like-, or classical-specific KLF5 peaks. See also Supplementary Data 6. (B-C) SLAM-seq analysis in AsPC-1 or T3M-4 following 4-hour total treatment with 300nM dTAG^{v}-1 or dTAG^{v}-1-NEG (control), including 2-hour 4sU labeling. Representative of 2 (T3M-4) or 3 (AsPC-1) biological replicates. Gene Set Enrichment analysis (GSEA) using gene sets comprised of all HOMER-annotated protein coding genes adjacent to classical- or basal-like-specific KLF5 peaks or regions of decreased H3K27ac signal following dTAG. Significance by GSEA. NES = normalized enrichment score. FWER = family-wise error rate. (D) HOMER Motif analysis of common KLF5 peaks. The top 4 TF family motifs within each peak set were selected. HOMER reported q-values. (E-F) KLF5, H3K27ac, and HNF4α (AsPC-1) or p63 (T3M-4) ChIP-seq. (E) Heatmap for KLF5 and HNF4α or p63 at all KLF5 peaks (MACS2 $q < 0.01$). Rows = 10Kb genomic regions centered on KLF5 peaks, ordered by KLF5 signal. Metagene plots (above) show the average signal for each factor across all peaks. (F) ChIP tracks at representative loci associated with classical (top) or basal-like (bottom) PDAC, visualized in UCSC genome browser. Scaled track heights indicated (right). Source data are provided as a Source Data file.

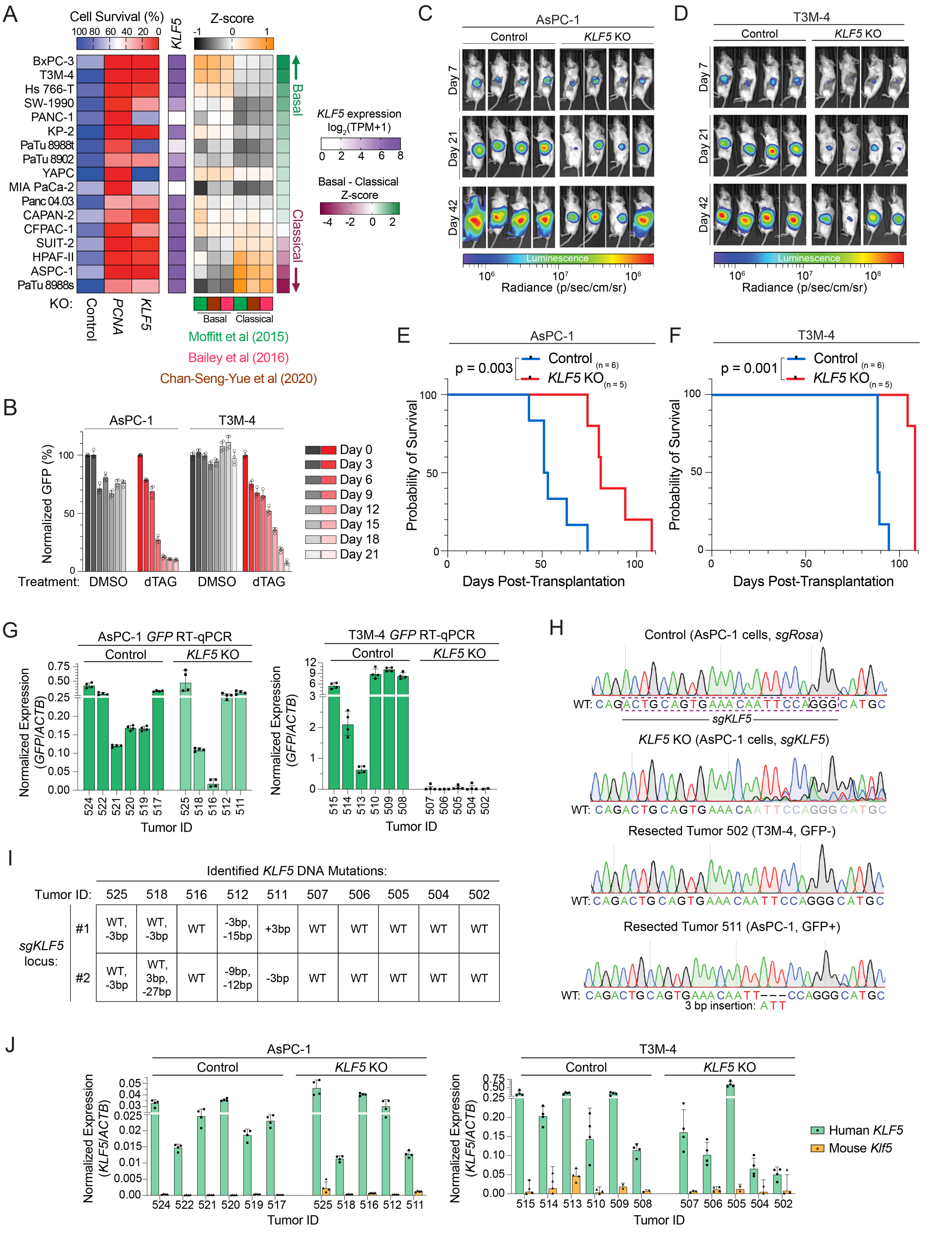

Supplementary Figure 5. KLF5 is a genetic dependency in classical and basal-like PDAC. (A) KLF5 dependency and expression (CCLE) in human PDAC cell lines. Heatmap (middle) shows *KLF5*

(log$_2$(*KLF5* TPM+1)). Heatmap (left) depicts competition-based fitness assays in Cas9-expressing PDAC cell lines following CRISPR-Cas9 knockout (KO) of *KLF5*, *PCNA* (lethal control), or *Rosa26* (negative control) using sgRNAs coupled to GFP. Heatmap color indicates the normalized GFP% (Day 21 %GFP+ normalized to day 3 post-transduction). Two independent sgRNAs per KO. n = 2-5 biological replicates (varied across cell lines, all replicates included in the Source Data). Heatmap (right) shows expression of genes corresponding to the listed basal-like- versus classical- PDAC identity signatures in PDAC cell lines (CCLE), using a Z-score analysis of variance-stabilized transformed counts. Scale bars indicate Z-scores. See also Supplementary Data 3. (B) Competition-based fitness assays of 1:1 pooled GFP-positive FKPB12^{F36V}-KLF5-expressing and GFP-negative parental AsPC-1 and T3M-4 cells treated with 400 nM dTAG^{v}-1 or DMSO (control). Data shown as mean ± SD of normalized %GFP (to day 0, start of treatment). n=4 biological replicates. (C-D) Representative bioluminescent images of orthotopic PDAC tumors. Pancreas-side of four representative mice in each group at indicated timepoints. Scale bar = log(Luminescence). (E-F) Mice were monitored following transplantation until humane endpoint based on tumor size or body condition. Evidence of advanced disease, including metastases, detected by necropsy in all 22 mice at endpoint. Significance evaluated by Log-rank test. (G, J) RT-qPCR analysis of (G) *GFP* transgene (coupled to sgRNAs in the lentiviral expression vector) and (J) *KLF5* in resected orthotopic PDAC tumors. Normalized expression values calculated as $2^{-\Delta Ct}$ normalized to the expression of *ACTB* in the same tumor sample. Each point represents a technical replicate (n=4 per tumor). Data shown as mean ± SD of technical replicates from one representative experiment. qPCR repeated twice (with independent primer pairs) with similar results. (H,I) Sanger sequencing of *sgKLF5* cut site from genomic DNA from resected tumors. (H) sgRNA recognition sequence is labeled with a purple dashed box. The canonical KLF5 DNA sequence is annotated below each track. (I) Description of the identified *KLF5* DNA mutations in each tumor sample. + = insertion, – = deletion, WT = canonical sequence. (J) Human *KLF5* and mouse *Klf5* expression values both normalized to human *ACTB*. Source data are provided as a Source Data file.

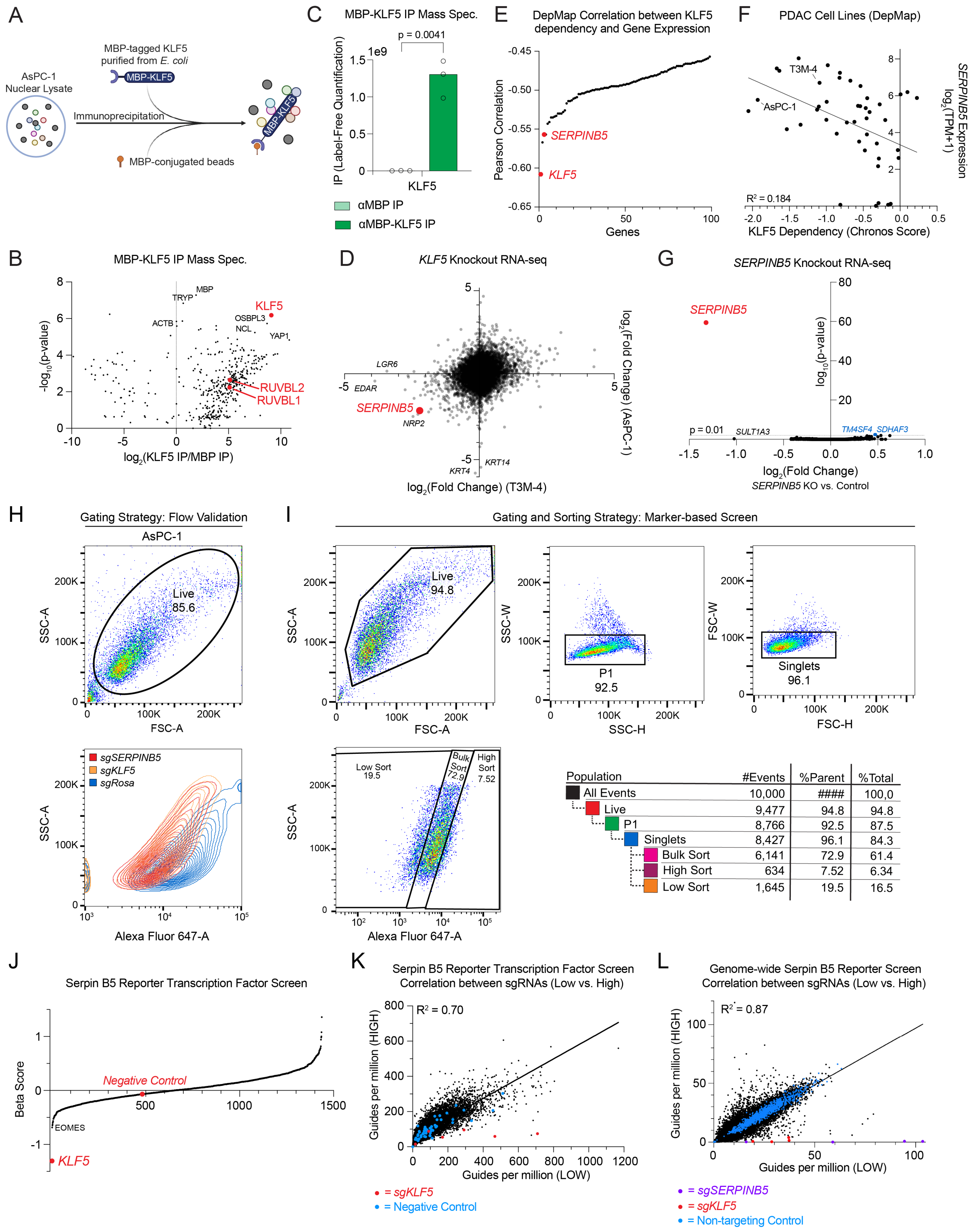

Supplementary Figure 6. Optimization of a FACS-based CRISPR screen to identify KLF5 coactivators. (A) Schematic of maltose-binding protein (MBP)-tagged KLF5 immunoprecipitation

(IP) mass spectrometry (MS). Created in BioRender. Cunniff, P. (2025) https://BioRender.com/9yws62k. (B-C) Detection of KLF5 interactors by pulldown-MS. MBP alone served as negative control. n = 3 independent IPs. See also Supplementary Data 8. (B) Scatterplot of protein enrichment in MBP-KLF5 versus MBP IPs. Each point is an individual protein. Significance calculated by unpaired two-tailed Student's t-test of log-transformed Label-free quantification (LFQ) versus empty lane background. (C) LFQ of peptides mapping to KLF5 in each IP. Significance assessed by unpaired two-tailed Student's t-test. (D,G) RNA-seq in AsPC-1, and T3M-4 on day 5 following CRISPR-Cas9 knockout (KO) of *KLF5* (D), *SERPINB5* (G), or *ROSA26* (control). Two independent sgRNAs used per KO. Fold change and significance calculated by DESeq2. See also Supplementary Data 4. (D) Log_2(Fold Change) following *KLF5* KO in T3M-4 (y-axis) vs. AsPC-1 (x-axis). n=4 (T3M-4) or 6 (AsPC-1) independent KO samples. (E-F) Correlation between KLF5 dependency and gene expression across 54 PDAC cell lines (DepMap) (E) Each point is an individual gene, ranked by Pearson correlation with KLF5 dependency. (F) Scatterplot of KLF5 Dependency versus *SERPINB5* expression. Each point is a cell line. R^2 was calculated by a simple linear regression (plotted). (G) Volcano plot of differentially expressed genes (DEGs) following *SERPINB5* KO. All DEGs ($p < 0.01$) labeled. (H) Flow cytometry plots depicting Serpin B5 staining of AsPC-1 PDAC cells transduced with sgRNAs targeting *ROSA26*, *KLF5*, and *SERPINB5*. SSC = side scatter, FSC = forward scatter. A = area. Associated with Figure 3B. (I) Gating and sorting strategy for Fluorescence-Activated Cell Sorting (FACS) of AsPC-1 PDAC cells for marker-based CRISPR screening. H = height, W = width. Associated with Figure 3D. (J-L) Serpin B5 reporter screens in AsPC-1. See also Supplementary Data 1. (J-K) Transcription factor-focused screen. (J) Beta scores and significance calculated by MAGeCK (maximum likelihood estimation). Negative beta scores indicate enrichment in the Serpin $B5^{low}$ population. Points = genes, ordered along the x-axis by β-score. Select outliers labeled. (K-L) sgRNA enrichment in the "High" and "Low" populations. Datapoints = sgRNAs. sgRNAs targeting *KLF5*, *SERPINB5*, or negative controls labeled. (L) Genome-wide Serpin B5 reporter CRISPR Screen. Source data are provided as a Source Data file.

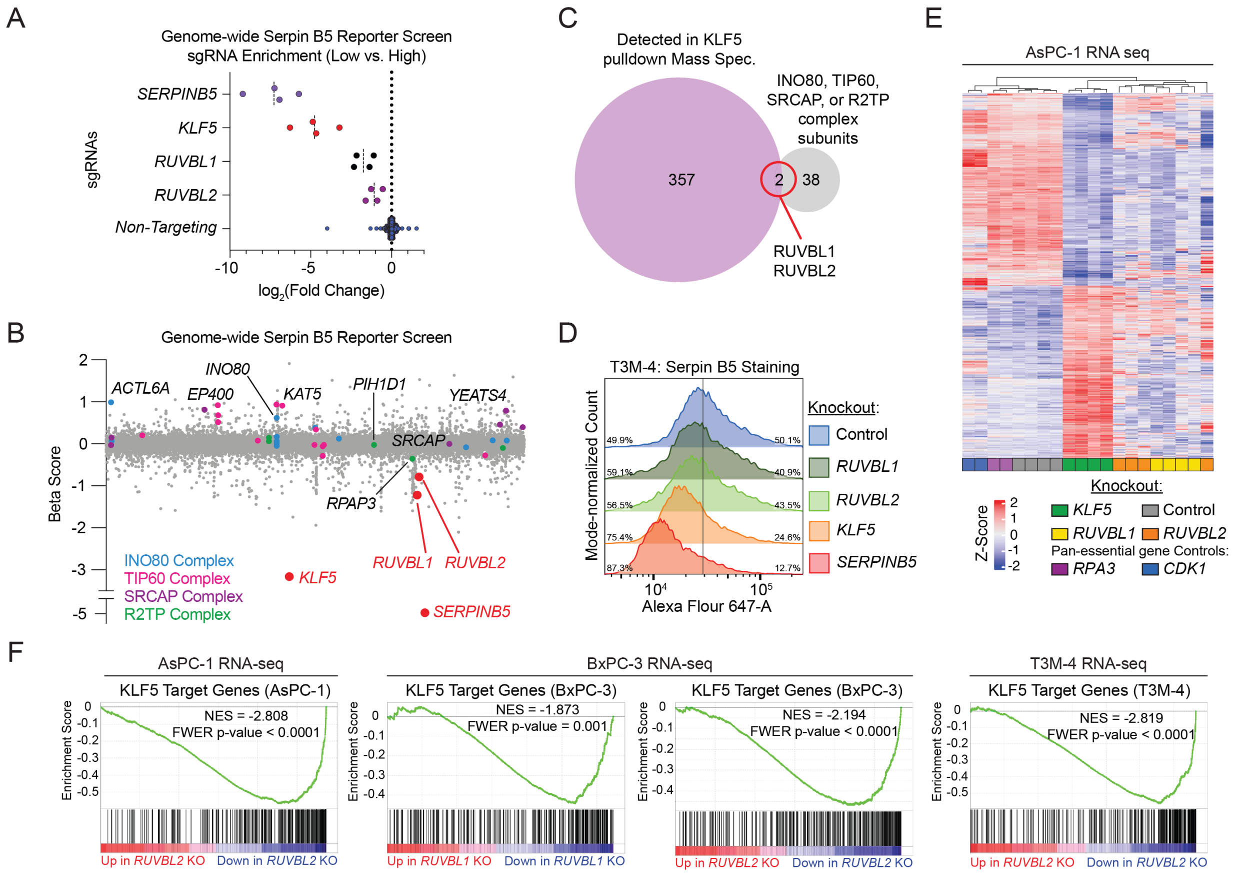

Supplementary Figure 7. Integrated proteomic and reporter screens identify RUVBL1 and RUVBL2 as KLF5 coactivators independent of INO80-family chromatin remodelers. (A-B) Genome-wide Serpin B5 reporter screen in AsPC-1. Negative beta scores or fold changes indicate enrichment in the Serpin B5^{low} population. See also Supplementary Data 1. (A) Datapoints = sgRNAs. Dashed line = mean. (B) Beta scores and significance calculated by MAGeCK (maximum likelihood estimation). Points = genes, ordered along the x-axis alphabetically. Members of the INO80, TIP60, SRCAP, and R2TP complexes are labeled. (C) Venn diagram showing overlap of enriched KLF5 interactors detected by Mass spectrometry (purple) with proteins in the INO80, TIP60, SRCAP, and R2TP complexes (grey). (D) Flow cytometry of T3M-4 cells methanol-fixed and stained with anti-Serpin B5 antibody 7 days following CRISPR-Cas9 knockout (KO) of *SERPINB5*, *KLF5, RUVBL1, RUVBL2*, or *ROSA26* (control). One representative of two independent sgRNAs per KO shown. Representative of 3 independent experiments. (E-F) RNA-seq in AsPC-1, BxPC-3, and T3M-4 on day 5 following KO of the indicated genes, or *ROSA26* (control). 2 independent sgRNAs were used for *KLF5*, *RUVBL1*, *RUVBL2*, and *ROSA26* KO. Fold change and significance of differentially expressed genes (DEGs) calculated by DESeq2. n = 2 biological replicates per sgRNA. (E) Heatmap of z-scored, variance-stabilized, normalized gene counts for the top 1000 DEGs following *KLF5* KO. Columns = samples, Rows = genes, clustered using Euclidian distance (dendrogram, top). Variance stabilized transformed counts calculated by DESeq2. (F) Gene Set Enrichment Analysis of DEGs following *RUVBL1* or *RUVBL2* KO. Significance calculated by GSEA. NES = Normalized Enrichment Score. FWER = Family-wise Error Rate. Source data are provided as a Source Data file.

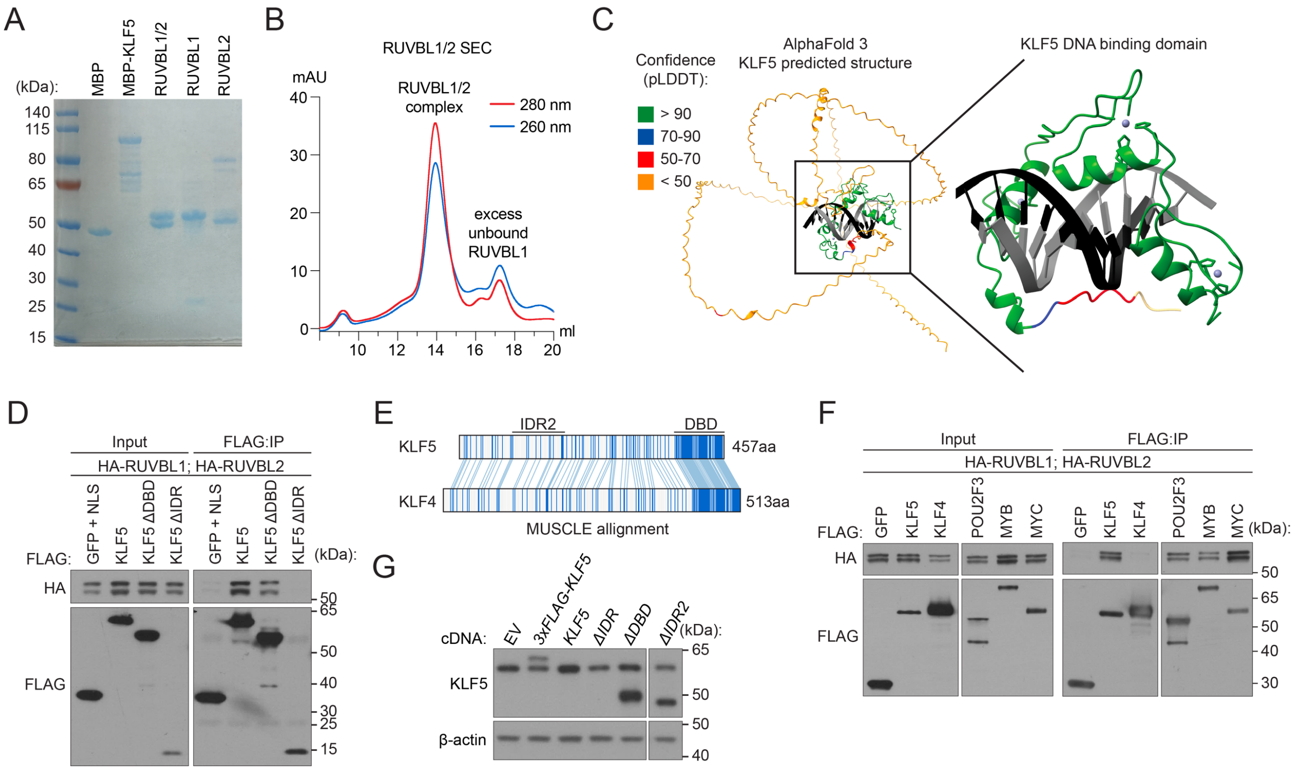

Supplementary Figure 8. KLF5 directly interacts with RUVBL1/2 through the KLF5 IDR2. (A-B) Recombinant MBP, MBP-tagged KLF5, RUVBL1, RUVBL2, and RUVBL1/2 purified from *E. coli*. Representative of 3 independent protein purifications. (A) Coomassie stain of 10µg each purified protein. (B) Size Exclusion Chromatography (SEC) with a Superose™ 6 Increase small-scale SEC column of the RUVBL1/2 hetero-complex. 280nm and 260nm emission profiles plotted. (C) AlphaFold3 predicted structure of KLF5 bound to DNA (grey). Predicted structure includes full-length KLF5, the complimentary DNA sequences GGCCCCACCCT and AGGGTGGGGCC, and three Zn^{2+} ions (purple). Colored according to the predicted local distance difference test (pLDDT) score for each amino acid in the final model. Zinc-coordinating cysteine and histidine amino acids are depicted. Prepared in ChimeraX-1.9. (D,F) Western blot following FLAG-KLF5 and HA-RUVBL1/2 co-immunoprecipitation. HA immunoblot detects RUVBL1/2 IP. 0.5% input loaded. FLAG, loading control. Representative of 2 (D) or 3 (F) biological replicates. (D) Amino acid compositions of the KLF5 deletion mutants are detailed in [Figure 4C]. (E) Multiple sequence alignment of KLF4 and KLF5 human protein sequences, performed by MUSCLE alignment using the Jalview-v2 software. KLF5 IDR2 and DNA Binding Domain (DBD) are labeled. (F-G) The blot was cropped to remove non-relevant lanes. Exposures are matched. (G) Protein expression of KLF5 and KLF5-mutant cDNAs in AsPC-1. cDNA expression constructs are labeled. KLF5 antibody detects both endogenous and ectopic KLF5 (cannot detect ΔIDR truncation mutant). β-actin, loading control. Representative of 2 independent experiments. Raw uncropped Western blots and Source Data are provided in the Source Data file.

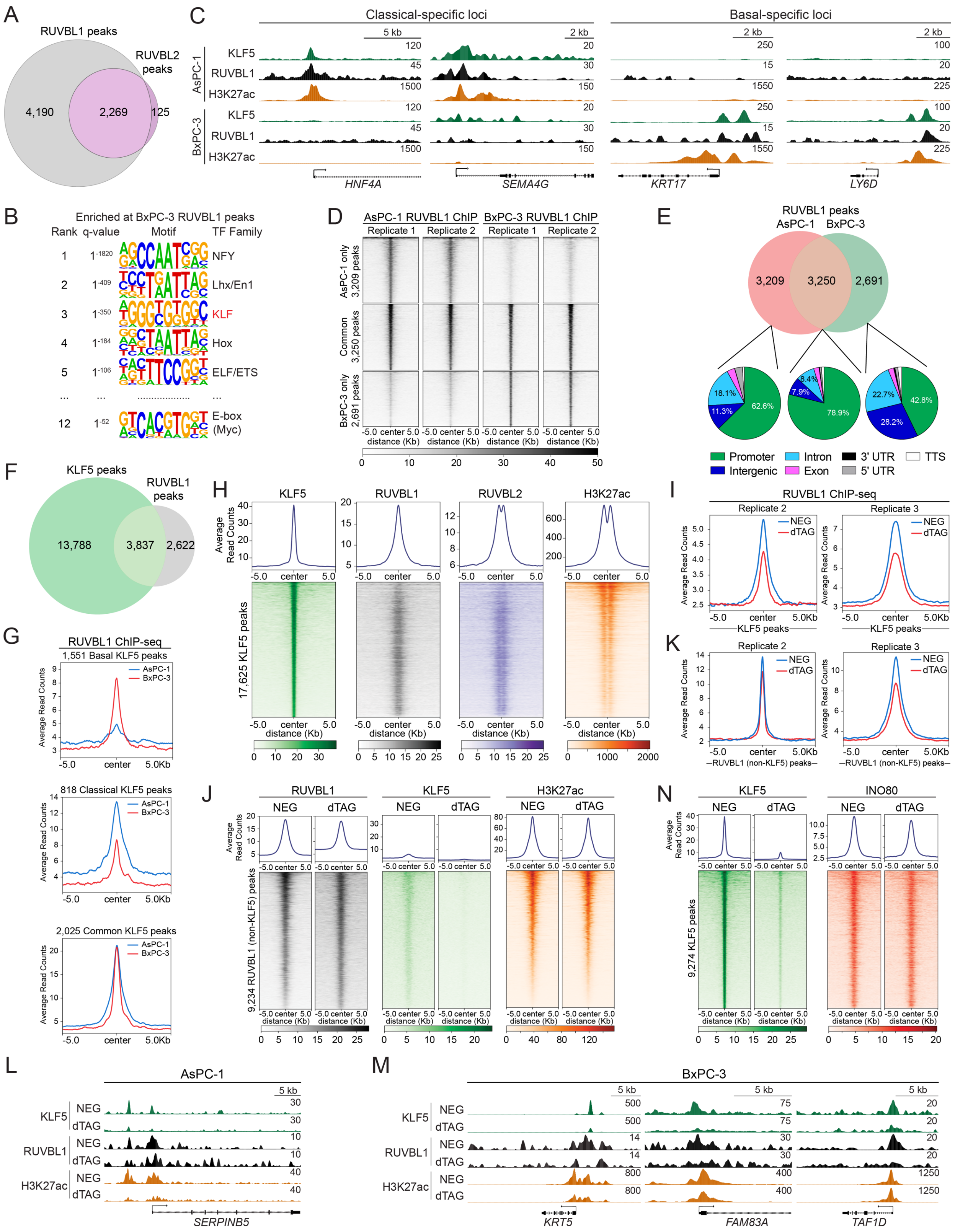

Supplementary Figure 9. KLF5 recruits RUVBL1/2 to chromatin in PDAC cells. (A,E,F) Venn diagrams of 6,459 RUVBL1 peaks, 2.394 RUVBL2 peaks, and 17,625 KLF5 peaks in AsPC-1 and

5,941 RUVBL1 peaks in BxPC-3 (MACS2 q < 0.01). Each is one representative of two independent IP replicates. (B) HOMER Motif analysis of RUVBL1 peaks in BxPC-3. The top 5 transcription factor family motifs were selected. See also Supplementary Data 7. (C, L-M) ChIP-seq tracks showing KLF5, RUVBL1, and H3K27ac enrichment at the indicated loci, visualized in UCSC genome browser. Matched, scaled track heights are listed (right). (D,H,J,N) Heatmaps for RUVBL1, KLF5, RUVBL2, H3K27ac, and INO80 ChIP-seq. Rows = 10Kb genomic regions centered on a (D,J) RUVBL1 or (H,N) KLF5 peak. (D-E) AsPC-1-specific, BxPC-3-specific, and Common RUVBL1 peaks were defined by a bedtools intersect analysis of RUVBL1 peaks (MACS2 q < 0.01). (E) HOMER annotations for RUVBL1 peaks in each region of the Venn diagram. (G) RUVBL1 ChIP-seq in AsPC-1 and BxPC-3. Classical, Basal-like, or Common KLF5 peaks are defined in Figure 2E. Metagene plots show the average signal for RUVBL1 across all peaks in each peak set, separated by cell line. (H) ChIP-seq in AsPC1. Regions are ordered by KLF5 signal, and this ordering is applied to all heatmaps. (H,J,N) Metagene plots (above) show the average signal for each factor across all peaks. (I-N) ChIP-seq analysis performed in (I-L,N) AsPC-1 or (M) BxPC-3 cells in which endogenous KLF5 is replaced with a FKBP12^{F36V}-KLF5, performed 3.5 hours following treatment with 300nM dTAG^{v}-1 (dTAG) or dTAG^{v}-1-NEG (NEG, control). Spike-in normalized using mouse chromatin (FC-1199). (I,K) Metagene plots show RUVBL1 ChIP-seq signal following treatment with dTAG or NEG, centered on (I) KLF5 or (K) RUVBL1 peaks in two additional replicates. (J,K) Only RUVBL1 peaks at sites without a KLF5 peak are included. (J,N) Regions are ordered by (J) RUVBL1 or (N) KLF5 signal in the NEG condition, and this ordering is applied to all heatmaps.

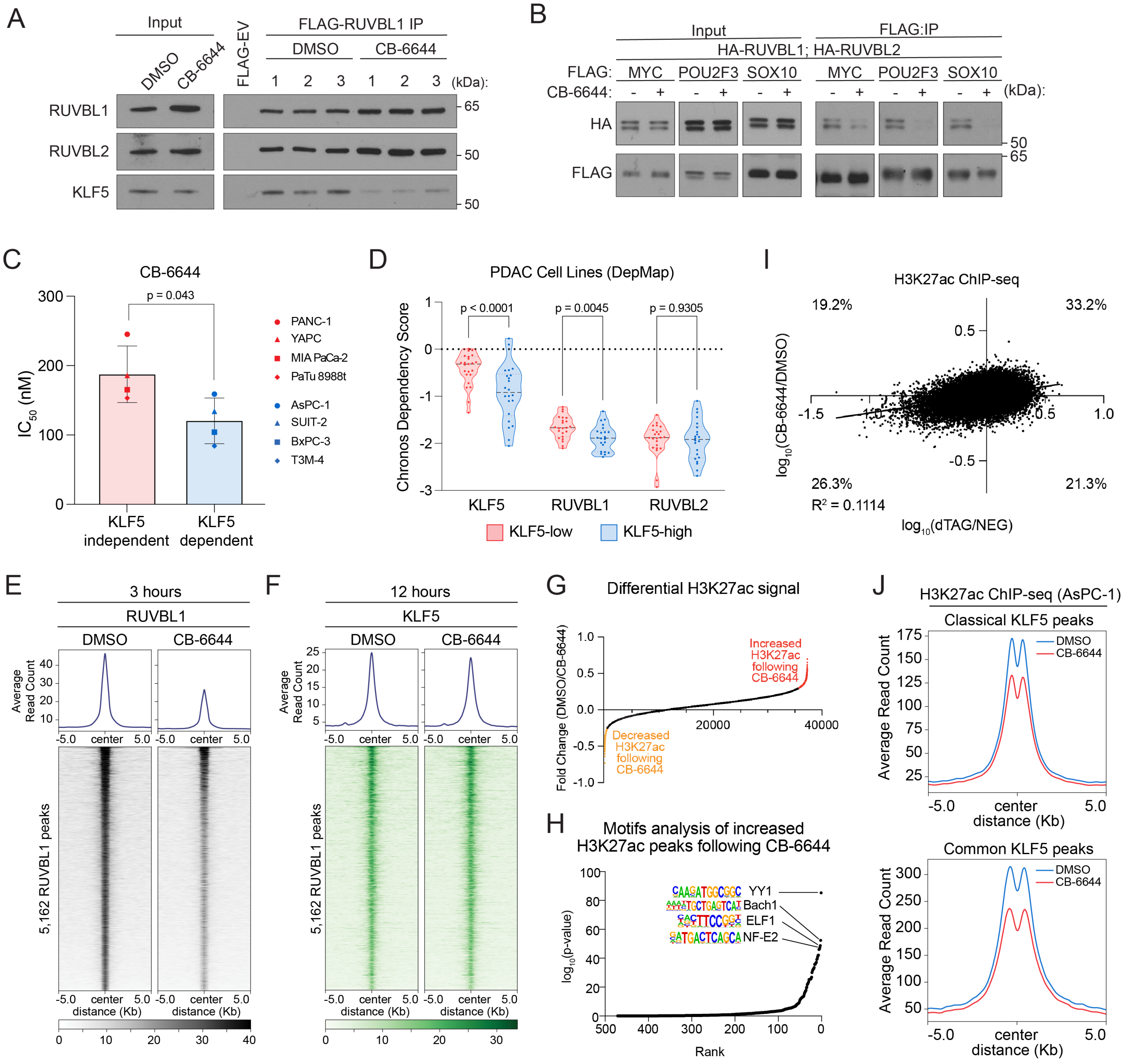

Supplementary Figure 10. Chemical inhibition of RUVBL1/2 ATPase disrupts its interaction with KLF5 on chromatin. (A) Western blot following FLAG-IP from AsPC-1 nuclear lysate following stable expression of FLAG-RUVBL1 cDNA by lentiviral transduction and 8-hour treatment with 750 nM CB-6644 or DMSO (control). 1% input loaded. Representative of two biological replicates. (B) Western blot following FLAG-TF and HA-RUVBL1/2 co-immunoprecipitation from HEK293T nuclear lysate. HA immunoblot detects RUVBL1/2 IP. 0.5% input loaded. FLAG immunoblot, loading control. Representative of two biological replicates. (C) CellTiter-Glo assay 72-96 hours following treatment with CB-6644 or DMSO in 8 human PDAC cell lines. Luminescence of each CB-6644 condition normalized to DMSO luminescence. Points = Average IC_{50} value for each cell line. Bars = mean ± SD. n = 7 biological replicates per cell line. Significance calculated by two-tailed student's t-test. (D) PDAC cell lines classified as KLF5-high or

KLF5-low according to *KLF5* expression (CCLE). KLF5, RUVBL1, and RUVBL2 Chronos scores for each cell line are plotted. Significance calculated by two-tailed Student's t-tests, no multiple comparison correction. n = 45 PDAC cell lines. (E,F) RUVBL1 and KLF5 ChIP-seq in AsPC-1 cells following 3- or 12-hour treatment with 750nM CB-6644 or DMSO. Heatmaps for RUVBL1 or KLF5 at all RUVBL1 peaks (MACS2 q<0.01). Rows = 10Kb genomic regions centered on RUVBL1 peaks, ordered by RUVBL1 signal (DMSO). Metagene plots (above) show average signal for each factor across all peaks. Spike-in normalized using mouse chromatin (FC-1199). (G) DiffBind analysis of global H3K27ac changes in AsPC-1 after CB-6644 (750 nM) vs. DMSO. DESeq2 assessed enrichment. Points = H3K27ac peaks, ranked by fold change. (H) HOMER motif analysis performed on loci with <0.25-fold H3K27ac increase. HOMER reported significance. Points = motifs, ranked by p-value. Selected motifs labeled. See also Supplementary Data 7. (I,J) H3K27ac ChIP-seq in AsPC-1 following 3.5-hour dTAGv-1, dTAGv-1-NEG (control), 12-hour CB-6644 or DMSO treatment. (I) Mean signal of each condition determined by averaging total signals ± 2kb from H3K27ac peak center. Differential H3K27ac signal at each locus was calculated by comparing average signals across conditions. Overall H3K27ac signal normalized across conditions prior to comparison. R^2 calculated by simple linear regression. See also Supplementary Data 10. (J) The metagene plot represents the average H3K27ac signal at each locus in the classical or common KLF5 peak sets. Source data are provided as a Source Data file.

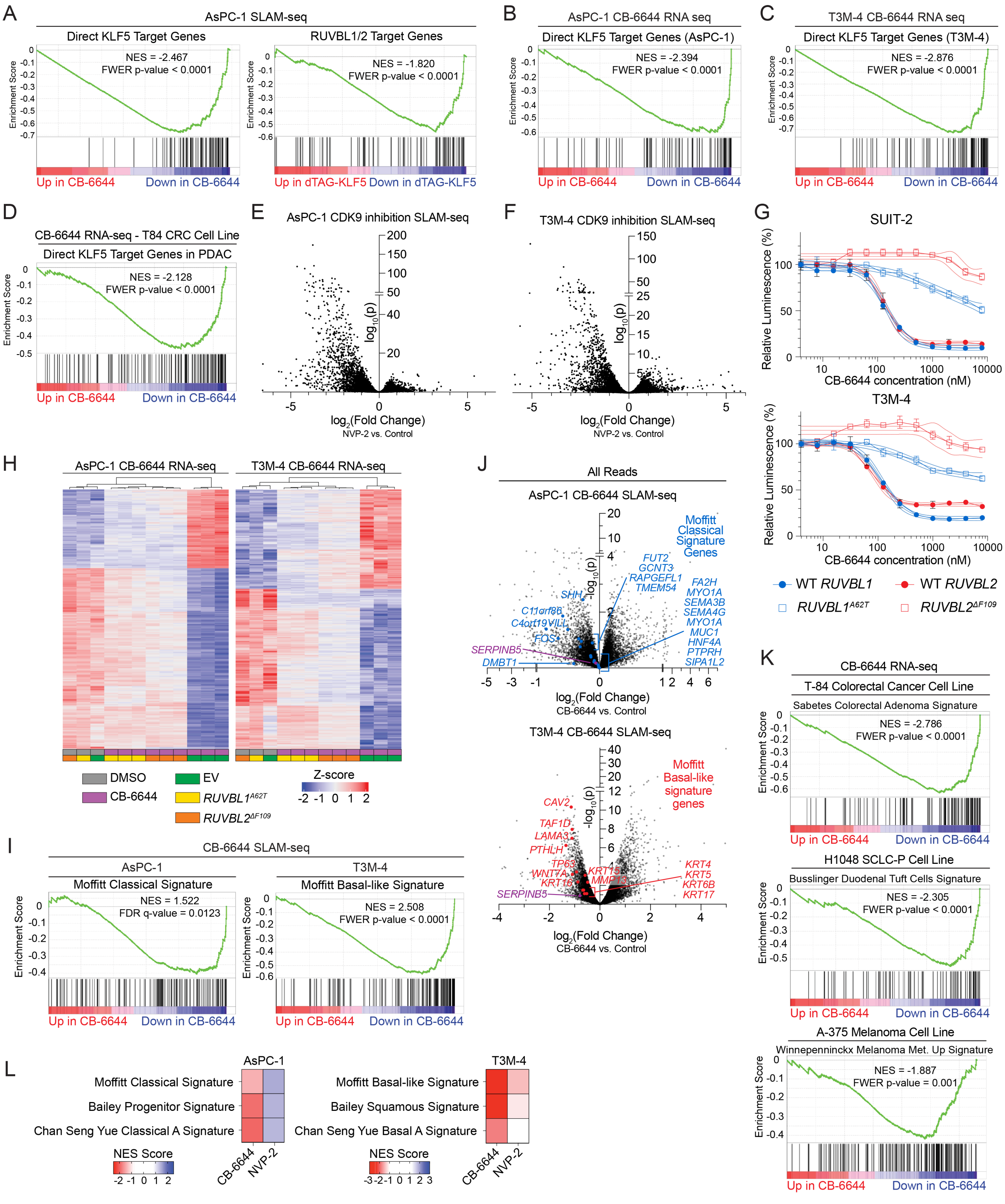

Supplementary Figure 11. Chemical inhibition of RUVBL1/2 ATPase suppresses KLF5-relevant transcriptional function. (A,E-F,I-J,L) SLAM-seq in AsPC-1 and T3M-4 cells following 4-hour total treatment with 300nM dTAG[v]-1, 300nM dTAG[v]-1-NEG (control), 750nM CB-6644, 750nM NVP-2, or DMSO (control), including 2-hour 4sU labeling. Representative of 2-3 biological

replicates. Fold change and significance of T→C converted transcripts following each respective treatment calculated with DESeq2. See also Supplementary Data 5. (A-D, I,K,L) Gene set enrichment analysis performed on the differentially expressed genes following CB-6644, NVP-2 or dTAG^{v}-1 treatment. Significance calculated by GSEA. NES = normalized enrichment score, FWER = family-wise error rate, Negative NES scores indicate that gene set is downregulated following treatment. (B-D,H,K) RNA-seq following 24-hour treatment with 750 nM CB-6644. Fold change and significance was calculated with DESeq2. See also Supplementary Data 9. (E-F) Volcano plots of all differentially expressed genes following NVP-2 treatment. (G-H) Prior to treatment, cDNAs encoding *RUVBL1*^{A62T}, *RUVBL2*^{dF109}, or empty vector (control) were stably expressed by lentiviral transduction. (G) Dose Response Curves (CellTiter-Glo assay) in PDAC cells treated with CB-6644 for 72-96 hours. Luminescence of each CB-6644 condition normalized to DMSO luminescence. Dot = mean normalized luminescence ± SD. n = 2. Lines = sigmoidal interpolation ± 95% confidence interval (H) Heatmap of z-scores of variance-stabilized normalized gene counts for the top 1000 differentially expressed genes between DMSO and CB-6644 (EV) for each cell line. Columns = samples, Rows = genes. Variance stabilized transformed counts were calculated with DESeq2. Samples were clustered using Euclidian distance (dendrogram, top). (J) Volcano plots of differentially expressed genes following CB-6644. Select Classical and Basal-like genes (Moffitt et al. 2015) and *SERPINB5* are labeled. All reads in the sequencing dataset are included in the analysis. (L) Heatmaps show NES values of the indicated PDAC lineage identity-specific gene sets following CB-6644 or NVP-2 treatment. Columns = treatments, rows = gene sets. Source data are provided as a Source Data file.

Supplementary Table 1. Sequences of Primers

RT-qPCR	
Primer	Sequence
ACTB_Hs_F	GTGCTACGTCGCCCTG
ACTB_Hs_R	GTTGGCGTACAGGTCTTTG
KLF5_1_Hs_F	CTATGTCAGCTGCCATGG
KLF5_1_Hs_R	GTTAAATTCTGGAGCATCTCTG
KLF5_2_Hs_F	CTTCATCTTTCTGTCCCTACCC
KLF5_2_Hs_R	CCATTGCTGCTGTCTGATTTG
GFP_F	GGACGACGGCAACTACAAGA
GFP_R	AAGTCGATGCCCTTCAGCTC
Actb_Mm_F	GCTGTGCTATGTTGCTCTAGAC
Actb_Mm_R	GTTGGCATAGAGGTCTTTACG
Klf5_Mm_F	GGCTCTCCCCGAGTTCACTA
Klf5_Mm_R	ATTACTGCCGTCTGGTTTGTC
Genomic DNA PCR	
Primer	Sequence
*KLF5*_site_1_F	CTTCATCTTTCTGTCCCTACCC
*KLF5*_site_1_R	GAGCATCTCTGCTTGTCTATCTG
*KLF5*_site_2_F	ATGGAGAAGTATCTGACACCTCAG
*KLF5*_site_2_R	ATTGTTCACCTCTGGAGC

Supplementary Table 2. Sequences of sgRNAs and dgRNAs

sgRNA	Protospacer Sequence
ROSA26 #1	AGTCGCTTCTCGATTATGGG
ROSA26 #2	GAAGATGGGCGGGAGTCTTC
RPA3	GATGAATTGAGCTAGCATGC
PCNA	GGACTCGTCCCACGTCTCTT
CDK1	ACTCAACTCCAGTTGACATT
KLF5 #1	TTGCACACCCCGCACTGGAA
KLF5 #2	TGTGTGCTTCCGGTAGTGGC
KLF5 #3	ACTGCAGTGAAACAATTCCA
KLF5 #4	GAAGAACTGGTCTACGACTG
SERPINB5 #1	AATGTTTCCCATACAGAACG
SERPINB5 #2	CTATGTGAAAAGGAGCCACT
RUVBL1 #1	ACTACTTACCAATGGCCCTG
RUVBL1 #2	ACTTGGATGTGGCTAATGCG
RUVBL1 #3	GAGACAGAGAATCCCATGGG
RUVBL2 #1	GATGATTGAGTCCCTGACCA
RUVBL2 #2	GGTGCCCACTCCCACATCCG
RUVBL2 #3	TGGCTGTGAATGGCGTGTCA
CHD4 #1	ATGTATGTCGTAACCTATGT
CHD4 #2	CAGTCACTCACCGTACAACG

dgRNA	Protospacer Sequence #1	Protospacer Sequence #2
KLF5 #3 | *KLF5* #4	ACTGCAGTGAAACAATTCCA	GAAGAACTGGTCTACGACTG

Supplementary Table 3: Antibodies

Antibody	Use	Source	Identifier
anti-KLF5<br>(Rabbit polyclonal)	Western blot (1:1,000),<br>ChIP-seq (7.5 µL/IP)	Abcam	Cat#ab137676;<br>RRID: AB_2744553
anti-RUVBL1<br>(Rabbit polyclonal)	Western blot (1:1,000),<br>ChIP-seq (10 µL/IP)	Thermo Fisher Scientific	Cat# PA5-29278,<br>RRID:AB_2546754
anti-RUVBL1<br>(Rabbit polyclonal)	Western blot (1:1,000)	Bethyl	Cat# A304-716A;<br>RRID:AB_2620911
anti-RUVBL2<br>(Rabbit polyclonal)	Western blot (1:800),<br>ChIP-seq (10 µL/IP)	ABclonal	Cat# A12564;<br>RRID:AB_2759406
anti-H3K27ac<br>(Rabbit polyclonal)	ChIP-seq (4µg/IP)	Abcam	Cat#: ab4729;<br>RRID:AB_2118291
anti-INO80<br>(Rabbit polyclonal)	Western blot (1:800),<br>ChIP-seq (10 µL/IP).	Proteintech	Cat#: 18810-1-AP;<br>RRID:AB_10598463
anti-HNF4a<br>(Rabbit monoclonal)	ChIP-seq (7.5 µL/IP)	Abcam	Cat#: ab181604;<br>RRID:AB_2890918
anti-p63-α (D2K8X) XP<br>(Rabbit monoclonal)	ChIP-seq (7.5 µL/IP)	Cell Signaling Technology	Cat#:13109;<br>RRID:AB_2637091
anti-Maspin (Serpin B5)<br>(Mouse monoclonal)	Western blot (1:500),<br>Flow cytometry (1:200)	Santa Cruz	Cat#: sc-271694;<br>RRID:AB_10714956
anti-TIP60<br>(Rabbit polyclonal)	Western blot (1:1,000)	Cell Signaling Technology	Cat#: 12058;<br>RRID:AB_2797811
anti-p400<br>(Rabbit polyclonal)	Western blot (1:1,000)	Abcam	Cat#: ab70301;<br>RRID:AB_1269644
anti-RPAP3<br>(Rabbit polyclonal)	Western blot (1:800)	Thermo Fisher Scientific	Cat#: PA5-58334;<br>RRID:AB_2646711
anti-SRCAP<br>(Rabbit polyclonal)	Western blot (1:500)	Thermo Fisher Scientific	Cat#: PA5-56012;<br>RRID:AB_2647912
anti-MBP<br>(Mouse monocloncal)	Western blot (1:5,000),<br>Immunoprecipitation<br>(7.5 µg/IP)	New England Biolabs	Cat#: E8032;<br>RRID:AB_1559730
anti-HA<br>(Rabbit polyclonal)	Western blot (1:2,000)	Sigma-Aldrich	Cat# H6908;<br>RRID:AB_260070
anti-FLAG M2<br>(Mouse monoclonal)	Western blot (1:2,500),<br>Immunoprecipitation<br>(35 µL bead slurry/IP)	Sigma-Aldrich	Cat#: F3165;<br>RRID:AB_259529
anti-β-Actin, HRP-linked<br>(Mouse monoclonal)	Western blot (1:20,000)	Sigma-Aldrich	Cat# A3854;<br>RRID:AB_262011
Goat anti-Mouse IgG (H+L) Polyclonal Cross-Adsorbed Secondary Antibody, Alexa Fluor™ 647	Flow cytometry (1:500)	Thermo Fisher Scientific	Cat#: A-21235;<br>RRID:AB_2535804
