# Supplementary material for: KLF5 enables dichotomous lineage programs in pancreatic cancer via the AAA+ ATPase coactivators RUVBL1 and RUVBL2
Source: Nat Commun. 2025 Nov 15;16:9996. doi: 10.1038/s41467-025-66007-0 (PMC12619835; doi:10.1038/s41467-025-66007-0)
Supplement: Supplementary file 2 — Description of Additional Supplementary Files [file 41467_2025_66007_MOESM2_ESM.pdf]

Descriptions of Additional Supplementary Files

Supplementary Data 1
Data from CRISPR Screens. Related to Figs. 1 and 3.

Supplementary Data 2
KLF5 expression in PDAC sequencing datasets. Related to Fig. 1.

Supplementary Data 3
PDAC Classification Signatures in human PDAC cell lines. Related to Fig. 1.

Supplementary Data 4
KLF5 Knockout RNA-sequencing data. Related to Fig. 2.

Supplementary Data 5
SLAM-seq data. Related to Figs. 2 and 5.

Supplementary Data 6
KLF5 ChIP-seq peaks. Related to Fig. 2.

Supplementary Data 7
Motif Enrichment Analysis Results. Related to Figs. 2, 4, and 5.

Supplementary Data 8
Mass Spectrometry Data. Related to Figs. 3.

Supplementary Data 9
RUVBL1/2 Knockout/Inhibition RNA-sequencing data. Related to Figs. 3 and 5.

Supplementary Data 10
dTAG- and CB-6644-sensitive cis-regulatory elements. Related to Fig. 5.

Supplementary Data 11
Gene Signatures.

Supplementary Data 12
Plasmids and cDNAs.
