# Supplementary material for: KLF5 enables dichotomous lineage programs in pancreatic cancer via the AAA+ ATPase coactivators RUVBL1 and RUVBL2
Source: Nat Commun. 2025 Nov 15;16:9996. doi: 10.1038/s41467-025-66007-0 (PMC12619835; doi:10.1038/s41467-025-66007-0)
Supplement: Supplementary file 15 — Reporting Summary [file 41467_2025_66007_MOESM15_ESM.pdf]

nature portfolio

Corresponding author(s): Christopher Vakoc

Last updated by author(s): Oct 9, 2025

Reporting Summary

Nature Portfolio wishes to improve the reproducibility of the work that we publish. This form provides structure for consistency and transparency in reporting. For further information on Nature Portfolio policies, see our Editorial Policies and the Editorial Policy Checklist.

Statistics

For all statistical analyses, confirm that the following items are present in the figure legend, table legend, main text, or Methods section.

n/a	Confirmed	
☐	☒	The exact sample size (*n*) for each experimental group/condition, given as a discrete number and unit of measurement
☐	☒	A statement on whether measurements were taken from distinct samples or whether the same sample was measured repeatedly
☐	☒	The statistical test(s) used AND whether they are one- or two-sided<br>*Only common tests should be described solely by name; describe more complex techniques in the Methods section.*
☒	☐	A description of all covariates tested
☐	☒	A description of any assumptions or corrections, such as tests of normality and adjustment for multiple comparisons
☐	☒	A full description of the statistical parameters including central tendency (e.g. means) or other basic estimates (e.g. regression coefficient) AND variation (e.g. standard deviation) or associated estimates of uncertainty (e.g. confidence intervals)
☐	☒	For null hypothesis testing, the test statistic (e.g. *F*, *t*, *r*) with confidence intervals, effect sizes, degrees of freedom and *P* value noted<br>*Give P values as exact values whenever suitable.*
☒	☐	For Bayesian analysis, information on the choice of priors and Markov chain Monte Carlo settings
☒	☐	For hierarchical and complex designs, identification of the appropriate level for tests and full reporting of outcomes
☐	☒	Estimates of effect sizes (e.g. Cohen's *d*, Pearson's *r*), indicating how they were calculated

Our web collection on statistics for biologists contains articles on many of the points above.

Software and code

Policy information about availability of computer code

Data collection	A MilliporeSigma® Guava easyCyte Flow Cytometer was used to acquire and analyze flow cytometry data for GFP competitions and gene complementation assays.<br>A BD Biosciences LSR Dual Fortessa flow cytometer was used for flow cytometry analysis.<br>A BD FACS ARIA-SORP II was used for flow cytometry-assisted cell sorting.<br>A Xenogen IVIS Spectrum imager was used to acquire bioluminescence data for orthotopic PDAC experiments.<br>A SoftMax Pro v6.3 (Molecular Devices) was used to measure luminescence.
Data analysis	Python 3.12, RStudio 2024.12.0+467, and GraphPad Prism v10.4.2 were used for data analysis.<br>Scanpy 1.7.2, Scrublet v0.2.3, and scikit-learn v0.22.2 were used for snRNA-seq analysis.<br>kallisto v0.45.0, tximport v1.36.0, rhdf5 v2.52.0, DESeq2 v1.48.0, and ComplexHeatmap v2.24.0 were used for RNA-seq analysis.<br>GSEA_v4.3.3 was used for Gene Set Enrichment Analysis.<br>slamdunk was used with default parameters for SLAM-seq analysis.<br>Bowtie2 v2.3.4.2, SAMtools v1.9, MACS2 v2.2.9.1, BEDTools v2.30.0, HOMER v5.1, and deepTools 3.5.2 were used for ChIP-seq analysis.<br>The UCSC Genome Browser was used to visualize ChIP-seq tracks.<br>Living Image Software (IVIS imaging systems) was used to analyze bioluminescence data for the orthotopic PDAC experiment.<br>ChimeraX-1.9 was used to analyze protein structures and generate figures.<br>FlowJo v10.6.2 was used to analyze flow cytometry data.

For manuscripts utilizing custom algorithms or software that are central to the research but not yet described in published literature, software must be made available to editors and reviewers. We strongly encourage code deposition in a community repository (e.g. GitHub). See the Nature Portfolio guidelines for submitting code & software for further information.

Data

Policy information about availability of data

All manuscripts must include a data availability statement. This statement should provide the following information, where applicable:

- Accession codes, unique identifiers, or web links for publicly available datasets
- A description of any restrictions on data availability
- For clinical datasets or third party data, please ensure that the statement adheres to our policy

All genomic datasets are available at the GEO database under accession codes GSE295347 (SLAM-seq; reviewer token: krglisqqtlsfjix), GSE295348 (CRISPR screens; reviewer token: kzcpewwovxizfgr), GSE295349 (RNA-seq; reviewer token: ibodsyccdxyrtwh), and GSE295354 (ChIP-seq; reviewer token: ypyhsmqgfjudxqr).
The hg38 human genome (UCSC) was used for all analyses.
The proteomic dataset is available at the PRIDE database under accession code PXD066112 (Reviewer Token: hr7welUpMFfk).

Research involving human participants, their data, or biological material

Policy information about studies with human participants or human data. See also policy information about sex, gender (identity/presentation), and sexual orientation and race, ethnicity and racism.

Reporting on sex and gender	No human participants or new human data was used in this study.
Reporting on race, ethnicity, or other socially relevant groupings	No human participants or new human data was used in this study.
Population characteristics	No human participants or new human data was used in this study.
Recruitment	No human participants or new human data was used in this study.
Ethics oversight	No human participants or new human data was used in this study.

Note that full information on the approval of the study protocol must also be provided in the manuscript.

Field-specific reporting

Please select the one below that is the best fit for your research. If you are not sure, read the appropriate sections before making your selection.

☒ Life sciences ☐ Behavioural & social sciences ☐ Ecological, evolutionary & environmental sciences

For a reference copy of the document with all sections, see nature.com/documents/nr-reporting-summary-flat.pdf

Life sciences study design

All studies must disclose on these points even when the disclosure is negative.

Sample size	Sample size calculations were based on previously published data. Figure legends indicate the sample sizes for each set of experiments. No statistical method was used to calculate sample size. All sample sizes were determined to be sufficient given that the differences among groups were consistent.
Data exclusions	For the Knockout RNA-seq analysis in T3M-4 (Figure 3), one RUVBL1 KO sample was excluded from analysis due to low library concentration resulting from ineffective library preparation.
Replication	The reported results were replicated across multiple experiments to generated reliable results and are described in more detail in the figure legends and methods section.
Randomization	For the orthotopic PDAC experiments, age-matched mice were randomly assigned to control or sgKLF5 groups. Orthotopic transplantations for control and treatment groups were performed on the same day. No other randomization was required in our experimental designs and thus not performed for any experiment.
Blinding	All experiments utilized standardized procedures and rely on objective measurements comparing control and treatment conditions, rather than subjective observations. For these reasons, blinding was both not possible in our experimental designs and would not bias the conclusions. Therefore, blinding was not performed for any experiment.

Reporting for specific materials, systems and methods

We require information from authors about some types of materials, experimental systems and methods used in many studies. Here, indicate whether each material, system or method listed is relevant to your study. If you are not sure if a list item applies to your research, read the appropriate section before selecting a response.

Materials & experimental systems

n/a	Involved in the study
☐	☒ Antibodies
☐	☒ Eukaryotic cell lines
☒	☐ Palaeontology and archaeology
☐	☒ Animals and other organisms
☒	☐ Clinical data
☒	☐ Dual use research of concern
☒	☐ Plants

Methods

n/a	Involved in the study
☐	☒ ChIP-seq
☐	☒ Flow cytometry
☒	☐ MRI-based neuroimaging

Antibodies

Antibodies used

The following antibodies were used in this study:
KLF5 (Rabbit polyclonal, Abcam, Cat#ab137676; RRID: AB_2744553; Lot: 1056810-1 and 1056810-7),
RUVBL1 (Rabbit polyclonal, Thermo Fisher, Cat# PA5-29278, RRID:AB_2546754; Lot: ZJ4490454A, ZA4162137 and AC4660180),
RUVBL2 (Rabbit polyclonal, ABclonal, Cat# A12564; RRID:AB_2759406; Lot: 0010460101),
INO80 (Rabbit polyclonal, Proteintech, Cat#: 18810-1-AP; RRID:AB_10598463; Lot: 00129808),
p63α (D2K8X-XP, Rabbit monoclonal, Cell Signaling Technology, Cat#:13109; RRID:AB_2637091; Lot: 39692S),
HNF4α (Rabbit monoclonal, Abcam, Cat#: ab181604; RRID:AB_2890918; Lot: 1084221-8)
H3K27ac (Rabbit polyclonal, Abcam, Cat#: ab4729; RRID:AB_2118291; Lot: 1035079-1 and GKR3216173-1),
RUVBL1 (WB only, Rabbit polyclonal, Bethyl, Cat# A304-716A; RRID:AB_2620911),
Maspin (Serpin B5) (Mouse monoclonal, Santa Cruz, Cat#: sc-271694; RRID:AB_10714956),
TIP60 (Rabbit polyclonal, Cell Signaling Technology, Cat#: 12058; RRID:AB_2797811)
p400 (Rabbit polyclonal, Abcam, Cat#: ab70301; RRID:AB_1269644),
RPAP3 (Rabbit polyclonal, Thermo Fisher, Cat#: PA5-58334; RRID:AB_2646711),
SRCAP (Rabbit polyclonal, Thermo Fisher, Cat#: PA5-56012; RRID:AB_2647912),
MBP (Mouse monoclonal, NEB, Cat#: E8032; RRID:AB_1559730),
HA (Rabbit polyclonal, Sigma-Aldrich, Cat# H6908; RRID:AB_260070),
FLAG-M2 (Mouse monoclonal, Sigma-Aldrich, Cat#: F3165; RRID:AB_259529),
β-Actin, HRP-conjugated (Mouse monoclonal, Sigma-Aldrich, Cat# A3854; RRID:AB_262011),
IgG (H+L), Alexa Fluor™ 647-conjugated (Goat anti-Mouse polyclonal, Thermo Fisher, Cat#: A-21235; RRID:AB_2535804),
KRT5 (Rabbit monoclonal, Cell Signaling Technology, Cat#: 25807; RRID:AB_2798912)

For Western blot, the following antibodies and dilutions were used: KLF5 (1:1,000). RUVBL1 (Thermo, 1:1,000), RUVBL1 (Bethyl, 1:1,000), RUVBL2 (1:800), Serpin B5 (1:500), HA (1:2,000), FLAG (1:2,500), β-Actin-HRP (1:20,000), TIP60 (1:1,000), INO80 (1:800), p400 (1:2,000), RPAP3 (1:800), SRCAP (1:500), MBP (1:5,000).

For ChIP-seq, the following antibodies and dilutions were used: KLF5 (7. 5μL/IP), RUVBL1 (10μL/IP), RUVBL2 (10μL/IP), INO80 (10μL/IP), p63α (7. 5μL/IP), HNF4α (7. 5μL/IP), and H3K27ac (4μg/IP).

For flow cytometry, the following antibodies and dilutions were used: Serpin B5 (1:200), and Alexa Fluor™ 647-conjugated goat anti-mouse (1:500).

For Immunoprecipitation-Mass Spec, 7.5μg of MBP antibody was used per IP.

Validation

The KLF5 (Abcam), RUVBL1 (Thermo), RUVBL1 (Bethyl), RUVBL2 (ABclonal), and Serpin B5 (Santa Cruz) antibodies were validated by CRISPR Knockout of each respective protein in human PDAC cell lines, followed by detection of the correct product via Western blot or flow cytometry. The KLF5, RUVBL1, and RUVBL2 antibodies were further validated by detection of the correct product via Western blot following cDNA overexpression and/or purification of recombinant protein from E. coli. The KLF5 antibody was further validated by response of FKBP12-F36V-KLF5 protein in human PDAC cell lines to dTAGv-1 treatment. The p63α (CST) antibody was previously validated by us in Maia Silva et al. 2024 through CRISPR knockout and detection of the correct product via Western blot. The H3K27ac (Abcam), INO80 (Proteintech), HNF4α (Abcam), TIP60 (CST), p400 (Abcam), RPAP3 (Thermo), SRCAP (Thermo), β-Actin (Sigma), FLAG-M2 (Sigma), HA (Sigma), and MBP (NEB) antibodies were validated by the vendors for human cell use and by us through Western blot detection of proteins at the correct molecular weight. INO80, TIP60, p400, RPAP3, and SRCAP antibodies were further validated by us through detection of each respective protein in RUVBL1 pulldown from human PDAC cells. The MBP antibody was further validated by us through detection of MBP-tagged purified proteins at the correct molecular weight. The FLAG-M2 and HA antibodies were further validated by us through immunoprecipitation assays using tagged overexpressed proteins of known size in human cancer cell lines.

Eukaryotic cell lines

Policy information about cell lines and Sex and Gender in Research

Cell line source(s)

The following cell lines used in this study were obtained from ATCC: HEK293T (Cat# CRL-3216; RRID:CVCL_0063), A-375 (female, Cat# CRL-1619; RRID:CVCL_0132), AsPC-1 (female, Cat# CRL-1682; RRID:CVCL_0152), BxPC-3 (female, Cat# CRL-1687; RRID:CVCL_0186), Capan-2 (male, Cat# HTB-80; RRID:CVCL_0026), CFPAC-1 (male, Cat# CRL-1918;

RRID:CVCL_1119), H1048 (female, Cat# CRL-5853; RRID:CVCL_1453), Hs 766-T (male, Cat# HTB-134; RRID:CVCL_0334), HPAF-II (male, Cat# CRL-1997; RRID:CVCL_0313), MIA PaCa-2 (male, Cat# CRL-1420; RRID:CVCL_0428), Panc 04.03 (male, Cat# CRL-2555; RRID:CVCL_1636), PANC-1 (male, Cat# CRL-1469; RRID:CVCL_0480), SW-1990 (male, Cat# CRL-2172; RRID:CVCL_1723), T84 (male, Cat# CCL-248; RRID:CVCL_0555).
The following cell lines used in this study were obtained from DSMZ: PaTu-8902 (female, Cat# ACC 179; RRID:CVCL_1845), PaTu-8988s (female, Cat# ACC 204; RRID:CVCL_1846), PaTu-8988t (female, Cat# ACC 162; RRID:CVCL_1847), YAPC (male, Cat# ACC 382; RRID:CVCL_1794).
The following cell lines used in this study were obtained from JCRB: KLM-1 (male, Cat#: RCB2138; RRID:CVCL_5146), KP-2 (female, Cat#: JCRB0181; RRID:CVCL_3004), SUIT-2 (male, Cat#: JCRB1094; RRID: CVCL_3172), SW-1990 (male, Cat# CRL-2172; RRID:CVCL_1723).
The murine FC-1199 pancreatic cancer cell line was generated in the Tuveson lab using tumor tissues from KrasG12DTrp53R172HPdx1-Cre (KPC) mice of a pure C57BL/6 genetic background.

Authentication

Cell lines were validated by the vendor (ATCC) using STR profiling and cell morphology analysis. Cell lines were further validated by STR profiling at an external facility after the establishment of Cas9 stable cell lines (Genetics core, University of Arizona). We verified that all human pancreatic cancer lines used in our study demonstrated the correct cell morphology throughout our studies.

Mycoplasma contamination

All cell lines are regularly verified to be mycoplasma contamination free.

Commonly misidentified lines
(See ICLAC register)

No ICLAC cell lines were used in this study.

Animals and other research organisms

Policy information about studies involving animals; ARRIVE guidelines recommended for reporting animal research, and Sex and Gender in Research

Laboratory animals

6-7 week old NSG (NOD.Cg-Prkdc scid Il2rg tm1Wjl /SzJ, Strain # 005557; RRID:IMSR_JAX:005557) were purchased from Jackson Laboratory. Mice ranging from 7 weeks old to 12 weeks old were enrolled in the orthotopic experiments. All mice were housed in specific pathogen-free facilities at CSHL under a 12h:12h light/dark cycle, with food and water available ad libitum. All purchased mice were acclimated to their facility for at least one week prior to enrollment in experiments.

Wild animals

No wild animals were included in this study.

Reporting on sex

All NSG mice included in this study were female.

Field-collected samples

No field-collected samples were included in this study.

Ethics oversight

All animal procedures and studies were approved by the Institute of Animal Care and Use Committee (IACUC) of CSHL and were conducted in accordance with the National Institutes of Health (NIH) Guide for the Care and Use of Laboratory Animals. All mice were euthanized using CO2 inhalation following institutional guidelines.

Note that full information on the approval of the study protocol must also be provided in the manuscript.

Plants

Seed stocks

No plants were used in this study.

Novel plant genotypes

No plants were used in this study.

Authentication

No plants were used in this study.

ChIP-seq

Data deposition

☒ Confirm that both raw and final processed data have been deposited in a public database such as GEO.

☒ Confirm that you have deposited or provided access to graph files (e.g. BED files) for the called peaks.

Data access links
May remain private before publication.

https://www.ncbi.nlm.nih.gov/geo/query/acc.cgi?acc=GSE295354

Files in database submission

fastq files and bigWig output from deepTools bamCoverage. bigWig output files are not included for input files: H3K27ac_AsPC1_rep1

Input_AsPC1_rep1
KLF5_AsPC1_rep1.1
KLF5_AsPC1_rep1.2
H3K27ac_BxPC3_rep1
Input_BxPC3_rep1
KLF5_BxPC3_rep1.1
KLF5_BxPC3_rep1.2
H3K27ac_HPAFII_rep1
Input_HPAFII_rep1
KLF5_HPAFII_rep1.1
KLF5_HPAFII_rep1.2
H3K27ac_T3M4_NEG_rep1.1
H3K27ac_T3M4_NEG_rep1.2
Input_T3M4_NEG_rep1
H3K27ac_T3M4_dTAG_rep1.1
H3K27ac_T3M4_dTAG_rep1.2
Input_T3M4_dTAG_rep1
KLF5_T3M4_dTAG_rep1
H3K27ac_T3M4_rep1
Input_T3M4_rep1
KLF5_T3M4_rep1.1
KLF5_T3M4_rep1.2
KLF5_T3M4_NEG_rep1
HNF4A_AsPC1_KLF5_HNF4A_overlap
Input_AsPC1_KLF5_HNF4A_overlap
KLF5_AsPC1_KLF5_HNF4A_overlap
H3K27ac_T3M4_KLF5_p63_overlap
Input_T3M4_KLF5_p63_overlap
KLF5_T3M4_KLF5_p63_overlap
p63_T3M4_KLF5_p63_overlap
H3K27ac_AsPC1_CB6644_12h_rep2
INO80_AsPC1_CB6644_12h_rep2
Input_AsPC1_CB6644_12h_rep2
RUVBL1_AsPC1_CB6644_12h_rep2
H3K27ac_AsPC1_DMSO_12h_rep2
INO80_AsPC1_DMSO_12h_rep2
Input_AsPC1_DMSO_12h_rep2
RUVBL1_AsPC1_DMSO_12h_rep2
INO80_AsPC1_dTAG_KLF5_for_INO80
Input_AsPC1_dTAG_KLF5_for_INO80
KLF5_AsPC1_dTAG_KLF5_for_INO80_rep1
KLF5_AsPC1_dTAG_KLF5_for_INO80_rep2
INO80_AsPC1_NEG_KLF5_for_INO80
Input_AsPC1_NEG_KLF5_for_INO80
KLF5_AsPC1_NEG_KLF5_for_INO80_rep1
KLF5_AsPC1_NEG_KLF5_for_INO80_rep2
H3K27ac_AsPC1_RUVBL12_overlap
H3K27ac_BxPC3_RUVBL12_overlap
Input_AsPC1_RUVBL12_overlap
Input_BxPC3_RUVBL12_overlap
KLF5_AsPC1_RUVBL12_overlap_rep1
KLF5_AsPC1_RUVBL12_overlap_rep2
KLF5_BxPC3_RUVBL12_overlap
RUVBL1_AsPC1_RUVBL12_overlap_rep1
RUVBL1_AsPC1_RUVBL12_overlap_rep2
RUVBL1_BxPC3_RUVBL12_overlap_rep1
RUVBL1_BxPC3_RUVBL12_overlap_rep2
RUVBL2_AsPC1_RUVBL12_overlap
H3K27ac_AsPC1_CB6644_3h
Input_AsPC1_CB6644_3h
KLF5_AsPC1_CB6644_3h_rep1
KLF5_AsPC1_CB6644_3h_rep2
RUVBL1_AsPC1_CB6644_3h
H3K27ac_AsPC1_DMSO_3h
Input_AsPC1_DMSO_3h
KLF5_AsPC1_DMSO_3h_rep1
KLF5_AsPC1_DMSO_3h_rep2
RUVBL1_AsPC1_DMSO_3h
H3K27ac_AsPC1_CB6644_12h_rep1
Input_AsPC1_CB6644_12h_rep1
KLF5_AsPC1_CB6644_12h_rep1
RUVBL1_AsPC1_CB6644_12h_rep1
RUVBL2_AsPC1_CB6644_12h_rep1
H3K27ac_AsPC1_DMSO_12h_rep1
Input_AsPC1_DMSO_12h_rep1
KLF5_AsPC1_DMSO_12h_rep1

RUVBL1_AsPC1_DMSO_12h_rep1
RUVBL2_AsPC1_DMSO_12h_rep1
H3K27ac_AsPC1_dTAG_KLF5_rep1
Input_AsPC1_dTAG_KLF5_rep1
KLF5_AsPC1_dTAG_KLF5_rep1
RUVBL1_AsPC1_dTAG_KLF5_rep1
H3K27ac_AsPC1_NEG_KLF5_rep1
Input_AsPC1_NEG_KLF5_rep1
KLF5_AsPC1_NEG_KLF5_rep1
RUVBL1_AsPC1_NEG_KLF5_rep1
H3K27ac_AsPC1_dTAG_KLF5_rep2
KLF5_AsPC1_dTAG_KLF5_rep2
RUVBL1_AsPC1_dTAG_KLF5_rep2
Input_AsPC1_dTAG_NEG_rep2
H3K27ac_AsPC1_NEG_KLF5_rep2
KLF5_AsPC1_NEG_KLF5_rep2
RUVBL1_AsPC1_NEG_KLF5_rep2
H3K27ac_AsPC1_dTAG_KLF5_rep3
KLF5_AsPC1_dTAG_KLF5_rep3
RUVBL1_AsPC1_dTAG_KLF5_rep3
Input_AsPC1_dTAG_NEG_rep3
H3K27ac_AsPC1_NEG_KLF5_rep3
KLF5_AsPC1_NEG_KLF5_rep3
RUVBL1_AsPC1_NEG_KLF5_rep3
H3K27ac_BxPC3_NEG_KLF5
Input_BxPC3_NEG_KLF5
KLF5_BxPC3_NEG_KLF5
RUVBL1_BxPC3_NEG_KLF5
H3K27ac_BxPC3_dTAG_KLF5
Input_BxPC3_dTAG_KLF5
KLF5_BxPC3_dTAG_KLF5
RUVBL1_BxPC3_dTAG_KLF5
H3K27ac_AsPC1_forDiffBind_DMSO_rep1.1
H3K27ac_AsPC1_forDiffBind_DMSO_rep1.2
Input_AsPC1_forDiffBind_DMSO_rep1
H3K27ac_AsPC1_forDiffBind_DMSO_rep2.1
H3K27ac_AsPC1_forDiffBind_DMSO_rep2.2
Input_AsPC1_forDiffBind_DMSO_rep2
H3K27ac_AsPC1_forDiffBind_CB6644_rep1.1
H3K27ac_AsPC1_forDiffBind_CB6644_rep1.2
Input_AsPC1_forDiffBind_CB6644_rep1
H3K27ac_AsPC1_forDiffBind_CB6644_rep2.1
H3K27ac_AsPC1_forDiffBind_CB6644_rep2.2
Input_AsPC1_forDiffBind_CB6644_rep2
H3K27ac_AsPC1_forDiffBind_CB6644_rep3.1
H3K27ac_AsPC1_forDiffBind_CB6644_rep3.2
Input_AsPC1_forDiffBind_CB6644_rep3
H3K27ac_AsPC1_forDiffBind_CB6644_rep4.1
H3K27ac_AsPC1_forDiffBind_CB6644_rep4.2
Input_AsPC1_forDiffBind_CB6644_rep4
H3K27ac_AsPC1_forDiffBind_CB6644_rep5.1
H3K27ac_AsPC1_forDiffBind_CB6644_rep5.2
Input_AsPC1_forDiffBind_CB6644_rep5
H3K27ac_AsPC1_forDiffBind_CB6644_rep6.1
H3K27ac_AsPC1_forDiffBind_CB6644_rep6.2
Input_AsPC1_forDiffBind_CB6644_rep6
H3K27ac_AsPC1_forDiffBind_CB6644_rep7.1
H3K27ac_AsPC1_forDiffBind_CB6644_rep7.2
Input_AsPC1_forDiffBind_CB6644_rep7

Genome browser session
(e.g. UCSC)

https://genome.ucsc.edu/s/pcunniff/AT_SERPINB5_fig3_hg38
https://genome.ucsc.edu/s/pcunniff/Fig2_4cell_lines_hg38
https://genome.ucsc.edu/s/pcunniff/Fig2_TFs_AsPC1_T3M4_hg38
https://genome.ucsc.edu/s/pcunniff/Fig4_AsPC1_dTAG_KLF5_hg38
https://genome.ucsc.edu/s/pcunniff/Fig4_BxPC3_dTAG_KLF5_hg38
https://genome.ucsc.edu/s/pcunniff/Fig5_RUVBL12_CB6644_AsPC1
https://genome.ucsc.edu/s/pcunniff/FigS6_RUVBL12_AsPC1_BxPC3_hg38

Methodology

Replicates

For KLF5 ChIP-seq analysis in two classical (AsPC-1 and HPAF-II) and two basal-like (BxPC-3 and T3M-4) PDAC cell lines, two independent IPs were performed for each cell line. Only peaks detected by MACS2 in both replicates were included for downstream analyses. For acute KLF5 degradation experiments in AsPC-1, three independent biological replicates were performed, each including KLF5, RUVBL1, and H3K27ac IPs. Results were validated across all three replicates, and data from replicates are included in the Extended Data Figures. For RUVBL1 ChIP-seq in AsPC-1 and BxPC-3, two independent IPs were performed for each cell line. Only

peaks detected by MACS2 in both replicates were included for downstream analyses. For RUVBL1/2 ChIP-seq analysis following CB-6644 in AsPC-1, two RUVBL1 and H3K27ac biological replicates were performed. For differential H3K27ac analysis following CB-6644, 14 (CB-6644) and (DMSO) IP replicates were performed together. All replicates were included for DiffBind analysis. Raw sequencing files and processed data files for ll replicates for all ChIP-seq experiments are included in the GEO database.

Sequencing depth

ChIP-seq libraries were single-end sequenced for 76 or 100 bp at a sequencing depth of ≥ 50 million raw reads (H3K27ac or input) or ≥ 30 million raw reads (KLF5, HNF4α, p63, RUVBL1, RUVBL2, INO80) per sample.

Antibodies

For ChIP-seq, the following antibodies and dilutions were used:
KLF5 (Rabbit polyclonal, Abcam, Cat#ab137676; RRID: AB_2744553; Lot: 1056810-1 and 1056810-7, 7. 5μL/IP),
RUVBL1 (Rabbit polyclonal, Thermo Fisher, Cat# PA5-29278, RRID:AB_2546754; Lot: ZJ4490454A, ZA4162137 and AC4660180, 10μL/IP),
RUVBL2 (Rabbit polyclonal, ABclonal, Cat# A12564; RRID:AB_2759406; Lot: 0010460101, 10μL/IP),
INO80 (Rabbit polyclonal, Proteintech, Cat#: 18810-1-AP; RRID:AB_10598463; Lot: 00129808, 10μL/IP),
p63α (D2K8X-XP, Rabbit monoclonal, CST, Cat#:13109; RRID:AB_2637091; Lot: 39692S, 7. 5μL/IP),
HNF4α (Rabbit monoclonal, Abcam, Cat#: ab181604; RRID:AB_2890918; Lot: 1084221-8, 7. 5μL/IP)
H3K27ac (Rabbit polyclonal, Abcam, Cat#: ab4729; RRID:AB_2118291; Lot: 1035079-1 and GKR3216173-1, 4μg/IP)

Peak calling parameters

Mappability of each sample was higher than 65%. For narrow peak (KLF5, RUVBL1, RUVBL2, HNF4a, p63, INO80) and broad peak (H3K27ac) calling, MACS2 default parameters were used.

Data quality

Only peaks nominated with FDR 0.01 by MACS2 were used in this study.

Software

Single end 76 or 100 base pair sequencing reads were mapped to the hg38 genome using Bowtie2 BEDTools v2.3.4.2 with default settings. MACS2 v2.2.9.1 was used to call peaks using input genomic DNA control. Annotation and motif analysis of ChIP-seq peaks was performed using HOMER v5.11 with default settings. To visualize genomic tracks, bigWig files were generated from sorted, indexed BAM files using the deepTools v3.5.2 bamCoverage function. Reads from single-end sequencing were extended based on sonication fragment size (300 base pairs). For experiments involving KLF5 degradation or RUVBL1/2 inhibition, sequencing reads were first mapped to the mm10 genome. Unaligned reads were then mapped to hg38 for downstream analysis. bigWig files were normalized according to the number of reads aligned to the mouse genome for each sample.
To define BED files of peaks and peak overlaps, MACS2 output narrowPeak or broadPeak files were merged using bedtools v2.30.0 intersect tools. Regions of high artifactual mapping to chromatin, "Blacklisted" regions, were removed from BED files using bedtools intersect prior to each analysis. Heatmaps and average chromatin occupancy metaplots were generated using the computeMatrix and plotHeatmap functions of deepTools, taking bigWig and BED files as input.

Flow Cytometry

Plots

Confirm that:

☒ The axis labels state the marker and fluorochrome used (e.g. CD4-FITC).

☒ The axis scales are clearly visible. Include numbers along axes only for bottom left plot of group (a 'group' is an analysis of identical markers).

☒ All plots are contour plots with outliers or pseudocolor plots.

☒ A numerical value for number of cells or percentage (with statistics) is provided.

Methodology

Sample preparation

PDAC cells were trypsinized, resuspended in serum-containing media, counted, washed in ice cold PBS, and fixed in -20C methanol at ≤ 10x106 cells/mL under gentle vortexing. Cells were stored in methanol at -20C for at least 2 days and up to 1 month. One day before flow cytometry analysis, cells were pelleted, washed 1x in FACS buffer (1% (w/v) ultrapure BSA, 0.5% (w/v) sodium azide, and 1 mM EDTA in magnesium and calcium-free PBS), and incubated overnight in primary antibody diluted in FACS buffer at 10x106 cell/mL, rotating at 4C. The next day, cells were pelleted, washed 2x with FACS buffer, and incubated for 2-hours in 1:500 secondary antibody diluted in FACS buffer at 10x106 cell/mL, rotating at 4C protected from light. After washing 2x in FACS buffer, cells were resuspended in FACS buffer at 10x106 cell/mL and subjected to flow cytometry analysis or sorting.

Instrument

A BD Biosciences LSR Dual Fortessa flow cytometer was used for flow cytometry analysis.
A BD FACS ARIA-SORP II was used for flow cytometry-assisted cell sorting (FACS) with the 633 nm laser.

Software

BD Biosciences FlowJo v10 software was used for all analyses of flow cytometry data.

Cell population abundance

Cell populations sorted by protein abundance were subsequently subjected to DNA extraction and library preparation.

Gating strategy

For flow cytometry-based CRISPR screens, side scatter area (SSC-A) plot vs forward scatter (FSC-A) area was used to separate live cells from debris and dead cells. The SSC height versus width and FSC height versus width were sequentially gated to separate single cells from aggregates. The total number of cells sorted per screen was a minimum of 5000x the size of the sgRNA library. Cells were sorted into three different populations, with the cells expressing the highest 10% and bottom 20% of AlexaFluor647 signal sorted into the marker-high and marker-low bins respectively.

☒ Tick this box to confirm that a figure exemplifying the gating strategy is provided in the Supplementary Information.
